# Cutting-edge genetics in obsessive-compulsive disorder

**DOI:** 10.12703/r/9-30

**Published:** 2020-12-23

**Authors:** Leonardo Cardoso Saraiva, Carolina Cappi, Helen Blair Simpson, Dan J Stein, Biju Viswanath, Odile A van den Heuvel, YC Janardhan Reddy, Euripedes C Miguel, Roseli G Shavitt

**Affiliations:** 1 Department & Institute of Psychiatry, Hospital das Clinicas HCFMUSP, Faculdade de Medicina, Universidade de Sao Paulo, Sao Paulo, SP, Brazil; 2 Columbia University Irving Medical Center, Columbia University, New York, NY, 10032, USA; 3 The New York State Psychiatric Institute, New York, NY, 10032, USA; 4 SA MRC Unit on Risk & Resilience in Mental Disorders, Department of Psychiatry & Neuroscience Institute, University of Cape Town, Cape Town, South Africa; 5 Molecular Genetics Laboratory, National Institute of Mental Health & Neurosciences (NIMHANS); Accelerator Program for Discovery in Brain disorders using Stem cells (ADBS) Laboratory, NIMHANS, Bangalore, India; 6 Amsterdam University Medical Centers, Vrije Universiteit Amsterdam, Department of Psychiatry, Department of Anatomy & Neuroscience, Amsterdam Neuroscience, Amsterdam, Netherlands; 7 Obsessive-Compulsive Disorder (OCD) Clinic, Department of Psychiatry, NIMHANS, Bangalore, India

**Keywords:** obsessive-compulsive disorder, genetics, genomics

## Abstract

This article reviews recent advances in the genetics of obsessive-compulsive disorder (OCD). We cover work on the following: genome-wide association studies, whole-exome sequencing studies, copy number variation studies, gene expression, polygenic risk scores, gene–environment interaction, experimental animal systems, human cell models, imaging genetics, pharmacogenetics, and studies of endophenotypes. Findings from this work underscore the notion that the genetic architecture of OCD is highly complex and shared with other neuropsychiatric disorders. Also, the latest evidence points to the participation of gene networks involved in synaptic transmission, neurodevelopment, and the immune and inflammatory systems in this disorder. We conclude by highlighting that further study of the genetic architecture of OCD, a great part of which remains to be elucidated, could benefit the development of diagnostic and therapeutic approaches based on the biological basis of the disorder. Studies to date revealed that OCD is not a simple homogeneous entity, but rather that the underlying biological pathways are variable and heterogenous. We can expect that translation from bench to bedside, through continuous effort and collaborative work, will ultimately transform our understanding of what causes OCD and thus how best to treat it.

## Introduction

Obsessive-compulsive disorder (OCD) is a neuropsychiatric disorder characterized by intrusive thoughts (obsessions) and repetitive behaviors (compulsions)^1^. With a lifetime prevalence of 2–3% and a typically chronic course^2^, OCD is associated with considerable role impairment^3^, reduced quality of life^4^, and morbidity^5^. Moreover, individuals with OCD have an elevated mortality risk independently of the effects of comorbidities^6^.

**Figure 1.**
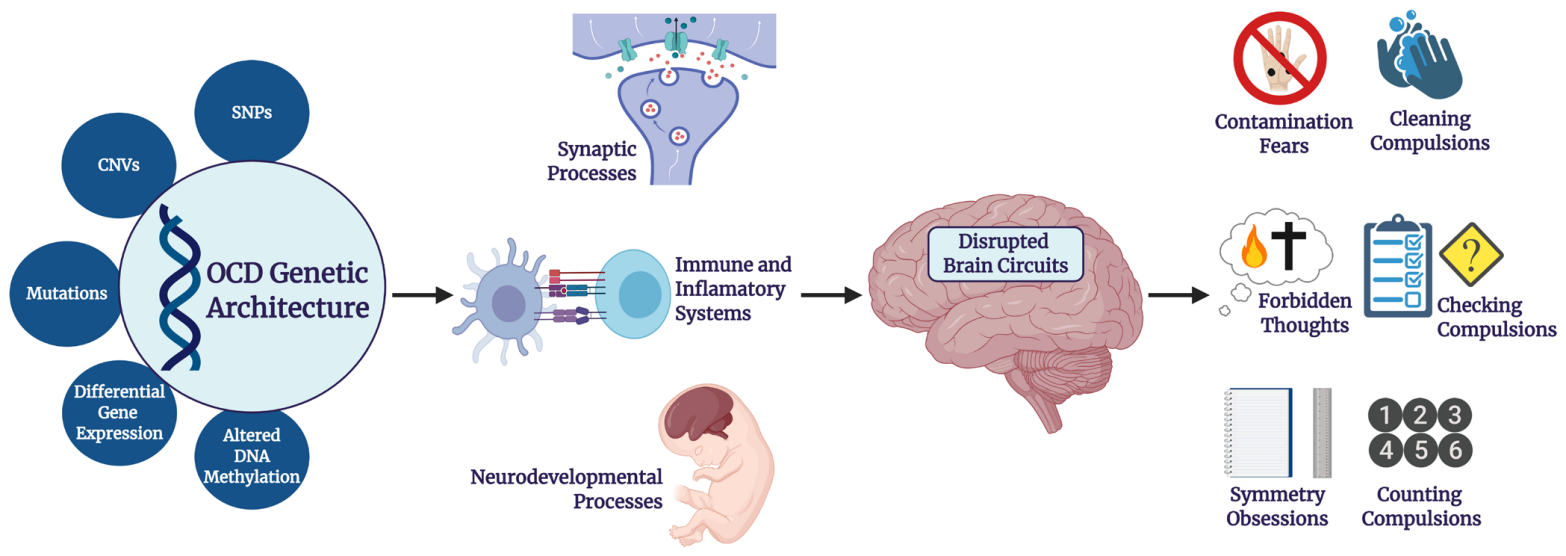
From genetic architecture to obsessive-compulsive disorder (OCD) symptomatology. The genetic architecture of OCD presumably underlies alterations in biological pathways, which in turn lead to disrupted brain circuits and OCD symptoms. CNV, copy number variant; SNP, single nucleotide polymorphism.

First-line treatments for OCD include both pharmacological and cognitive–behavioral approaches^7^; alone or combined, these can help about half of patients to achieve minimal symptoms^8^. The development of more targeted and effective treatment interventions may benefit from further understanding of the underlying etiology of OCD, including underlying genetic mechanisms. Here we review recent advances in OCD genetics research, including genome-wide association studies (GWAS), whole-exome sequencing (WES) studies, copy number variation (CNV) studies; gene expression, polygenic risk score (PRS), gene–environment interactions, experimental animal systems, human cell models, imaging genetics, pharmacogenetics, and studies of endophenotypes. We suggest that recent work converges in pointing out that genes involved in synaptic transmission, neurodevelopment, and the immune and inflammatory systems are involved in the pathophysiology of OCD (Figure 1). Given the genetic overlap between OCD and related disorders, further work is needed to assess the specificity of this involvement.

## From genetic epidemiology to genetic architecture

Family and twin studies are useful for exploring genetic and environmental contributions to a disease. In this context, familiality refers to increased clustering of a disorder amongst families^9^. Consistent with multiple previous studies^10–12^, a recent family study in Sweden provided further evidence for the familiality of OCD, especially when associated with tics^13^. Heritability can be defined as the proportion of variance in a phenotypic trait attributable to additive genetic effects (i.e. narrow-sense heritability, *h*^2^) or to total genetic effects (i.e. broad-sense heritability, *H*^2^)^14^. Henceforth in this article, narrow-sense heritability (*h*^2^) will be referred to as heritability. Past twin studies have estimated the heritability of OCD to range from 27 to 65%^15^, and a recent heritability estimate of 74% was reported for obsessive-compulsive traits in a pediatric nonclinical twin cohort^16^.

The heritability of OCD encourages further work on the genetics of this disorder, with the hope that this will, in turn, contribute to the development of more precise diagnostic and therapeutic approaches^17^. The term “genetic architecture” refers to the overall number, effect size, population frequency, and interactions of genetic variants associated with a phenotype^18^. Genetic studies that contribute to elucidating genetic architecture include GWAS, WES studies, CNV studies, gene expression analysis, gene–environment interaction studies, experimental models with animal and human cells, imaging genetics studies, pharmacogenetic studies, and studies of endophenotypes. We consider each in turn, presenting a summary of all of the cited studies after the section describing an exploratory model of the genetic architecture of OCD (Table 1).

**Table 1.** Summary of genetic studies in obsessive-compulsive disorder (OCD) cited throughout this review.

Author	Sample	Methodology	Findings
**Brander *et al*.** **(2019)**	4,085,367 individuals, including 22,232 OCD probands	Family study	Familial clustering of OCD, especially tic-related OCD. Using hazard ratios adjusted for sex and birth year, the risk of OCD in full siblings of patients with tic-related OCD and patients with non-tic-related OCD was estimated at 10.63 and 4.52, respectively.
**Burton *et al*.** ** (2018)**	220 pairs of non-clinical twins	Twin study	Heritability of obsessive-compulsive traits was estimated at 74%. Shared genetic factors explained most of the variance shared among the obsessive-compulsive trait dimensions examined in this study, which included cleaning/contamination, symmetry/ordering, rumination, superstition, counting/checking, and hoarding.
**Stewart *et al*.** ** (2013)**	1,465 OCD probands and 5,557 control subjects	Genome-wide association study (GWAS)	No genome-wide significant single nucleotide polymorphism (SNP) was detected in case- control analysis, which yielded the top SNPs located within *DLGAP1*. Trio-based analysis revealed a SNP reaching genome-wide significance located near *BTBD3*. Combining the two analyses revealed significant enrichment for regions regulating gene expression and methylation in the brain.
**Mattheisen *et al*.** ** (2015)**	1,406 OCD probands and 5,061 control subjects	GWAS	No genome-wide significant SNP was detected. Top-ranked SNPs were near *PTPRD*, *CDH9*, and *CDH10*. Two SNPs near *GRIK2* and *HACE1* detected in the first OCD GWAS were also among the significant SNPs.
**Den Braber** ***et al*. (2016)**	Obsessive-compulsive symptoms of 8,267 subjects	GWAS	A genome-wide significant SNP was detected at *MEF2BNB*. Gene-based testing revealed four genes significantly associated with obsessive-compulsive symptoms located in the 19p13.11 chromosomal region, which has been associated with brain and immune processes.
**Costas *et al*.** ** (2016)**	370 OCD probands and 443 control subjects	GWAS	No genome-wide significant SNP was detected. A polygenic risk score (PRS) estimated using the Psychiatric Genetics Consortium (PGC) schizophrenia (SCZ) meta-analysis sample significantly predicted OCD case-control status among the study sample.
**Guo *et al*.** ** (2017)**	2,998 OCD case-control samples and 6,898 autism spectrum disorder (ASD) case-control samples	GWAS	No genome-wide significant SNP was detected. Top-ranked SNP identified is in linkage disequilibrium (LD) with *TUBB3*. A PRS estimated using the ASD case-control sample significantly predicted case-control status in the OCD sample.
**PGC OCD** ** workgroup (2018)**	2,688 OCD probands and 7,037 control subjects	GWAS	No genome-wide significant SNP was detected. Most significant SNPs were in LD with *GRID2* and *KIT*. PRSs estimated based on the first and second OCD GWASs significantly predicted case-control status in the second and the first OCD GWASs case-control samples, respectively. SNP-based heritability was estimated at 0.28.
**Khramtsova *et al*.** ** (2018)**	PGC-OCD GWAS sample	GWAS	SNP-based heritability estimates were similar for male and female OCD, and significant genetic correlation between them was reported. *GRID2* and *GRP135* were associated only with female OCD in gene-based tests. SNPs with sex-specific effects were significantly enriched for regions regulating gene expression in brain and immune tissues.
**Smit *et al*. (2020)**	PGC-OCD GWAS sample and compulsive symptoms of 8,267 subjects	GWAS	No genome-wide significant SNP was detected. Significant genetic correlation between OCD and compulsive symptoms was found. *WDR7*, *ADCK1*, *GRID2*, and *KIT* were detected in gene-based tests. Top genes detected in gene-based tests were significantly enriched for genes expressed in the anterior cingulate cortex, nucleus accumbens, and amygdala.
**Cross-Disorder ** **Group of the ** **PGC (2019)**	232,964 probands diagnosed with anorexia nervosa (AN), attention deficit hyperactivity disorder (ADHD), ASD, bipolar disorder (BD), major depressive disorder (MDD), OCD, SCZ, or Tourette’s syndrome (TS) and 494,162 controls subjects	GWAS	Genetic correlation between OCD and AN was among the highest observed. Exploratory factor analysis suggested shared genetic liability among OCD, AN, and TS.
**Bralten *et al*.** ** (2020)**	650 non-clinical children and adolescents	GWAS	The individual results from the GWAS of obsessive-compulsive symptoms in the sample of 650 non-clinical children and adolescents were not reported owing to insufficient power. Nonetheless, PRS analysis revealed shared genetic etiology among OCD, obsessive- compulsive symptoms, and insulin signaling-related traits.
**Cappi *et al*. ** **(2016)**	17 OCD parent–child trios	Whole-exome sequencing study	20 nonsynonymous *de novo* single nucleotide variants (SNVs) detected among 17 OCD probands. SNVs were enriched in pathways related to the immune system and neurodevelopment.
**Cappi *et al*. ** **(2019)**	184 OCD and 777 control parent– child trios	Whole-exome sequencing study	A significantly higher prevalence of *de novo* damaging variants (33.9%) was found among probands. These variants were estimated to be present in 22.2% of overall OCD cases. *SCUBE1* and *CHD8* were identified as high-confidence risk genes for OCD. Genes carrying these variants significantly overlap among ASD and TS probands and were associated with immune system-related processes.
**Gazzellone *et al*. ** **(2016)**	307 pediatric OCD probands and 3,861 control subjects	Copy number variants study	A similar rate of rare copy number variants (CNVs) in early onset OCD probands and controls was observed. In 5.9% of probands, structural variants were detected in genes associated with brain function. Particularly, CNVs were found in three targets of fragile X mental retardation protein (FMRP), including *DLGAP1* and *PTPRD*.
**Grünblatt *et al.*** **(2017)**	121 pediatric OCD probands and 123 control subjects	Copy number variants study	Although no significant difference in number and size of rare CNVs between pediatric OCD patients and controls was detected, the number of CNVs encompassing genes involved in neurological function was higher among the former.
**Zarrei *et al*.** ** (2019)**	2,691 probands diagnosed with OCD, SCZ, ASD, or ADHD and 1,769 family members	Copy number variants study	Clinically relevant CNVs were found in 5.6% of OCD probands. Multiple brain-expressed genes impacted by CNVs in at least two probands were found. An increased burden of rare CNVs impacting genes involved in genomic stability was found in probands.
**Wang *et al*. ** **(2018)**	77 OCD, 39 MDD, and 40 SCZ probands and 67 control subjects	Gene expression analysis	A blood-based gene expression panel (including *FKBP1A*, *COPS7A*, *FIBP*, *TP73-AS1*, *SDF4*, and *GOLGA8A*) was able to distinguish OCD probands from MDD and SCZ probands and healthy controls with 88% sensitivity and 85% specificity.
**Piantadosi *et al*. ** **(2019)**	Postmortem brain samples of 8 OCD probands and 8 control subjects	Gene expression analysis	Overall reduced expression of genes related to excitatory synapses in the orbitofrontal cortex (OFC), but not in striatal regions, of OCD probands. Minor alterations were observed in the expression of genes related to inhibitory synapses in the OFC of OCD probands.
**Nissen *et al*. ** **(2016)**	21 pediatric and adolescent OCD probands and 12 female controls	DNA methylation analysis	No significantly different DNA methylation levels in candidate genes previously implicated in OCD were found between probands and controls.
**Yu *et al*. (2016)**	65 pediatric OCD probands and 96 control subjects	DNA methylation analysis	Multiple genes with different DNA methylation levels among OCD cases and controls were found. Further enrichment analysis implicated those genes in cell adhesion- and actin-related processes.
**Alemany-Navarro** ***et al*. (2019)**	103 OCD probands	Gene–environment interaction	Although not predicting treatment response, a PRS estimate based on OCD risk variants predicted pretreatment symptom severity.
**Mahjani *et al*.** ** (2020)**	822,843 individuals, including 7,184 probands diagnosed with OCD	Maternal effects	Genetic maternal effects (i.e. the influence of the maternal genotype on the phenotype of the offspring) accounted for 7.6% of the liability for OCD, whereas additive genetic effects accounted for 35%.
**Trankner *et al*.** ** (2019)**		Experimental animal system	Targeted deletion of Hoxb8-lineage microglia led to significant grooming and anxiety- like behaviors and stress-response among female rodents, which were ameliorated by suppressing female sex hormones.
**Delgado-** **Acevedo** ***et al*. (2018)**		Experimental animal system	Overexpression of EAAT3 in the frontal cortex, hippocampus, and striatum increased anxious and grooming behaviors and prolonged spontaneous recovery of conditioned fear among rodents. Moreover, alterations in NMDA receptor constitution and corticostriatal synaptic plasticity were observed.
**Zike *et al*. (2017)**		Experimental animal system	Constitutive reduced expression of EAAT3 in rodents caused dampened stereotyped response following dopaminergic challenges, which was rescued by restoration of EAAT3 expression in the midbrain.
**Krabbe *et al*. ** **(2017)**		Experimental animal system	Rodent with microglia-restricted progranulin (PGRN) inactivation exhibited increased self- grooming and marble-burying behaviors, which were normalized with suppression of nuclear factor κB (NF-κB) signaling.
**Ullrich *et al*.** ** (2018)**		Experimental animal system	*SPRED2* knockout mice displayed severe grooming and anxiety behaviors, dysfunctional thalamo-amygdala synapses, and altered expression of synaptic proteins in the amygdala. Inhibition of overactive (TrkB)/ERK-MAPK signaling ameliorated pathologic behavior.
**Van de ** **Vondervoort *et al*.** **(2020)**		Experimental animal system	TALLYHO/JngJ rodents, which are a model of human type 2 diabetes mellitus, exhibited increased compulsive and anxious behaviors in addition to structural brain abnormalities in midline corpus callosum, dorsomedial striatum, and superior cerebellar peduncles.
**Noh *et al*. (2017)**	592 OCD probands and 560 control subjects	Functional variants prioritization	Analysis of 608 genes potentially associated with OCD in human, rodent, and dog studies revealed significant functional variant burden in four genes (*NRXN1*, *HTR2A*, *CTTNBP2*, and *REEP3*) involved in brain pathways implicated in OCD. *NRXN1* achieved genome-wide significance after inclusion of GWAS data from 33,370 controls.
**Rodriguez *et al*.** ** (2017)**	102 OCD early onset OCD probands and 47 control subjects	Human cell models	Higher percentages of total monocytes and CD16^+^ monocytes in OCD probands as compared to control subjects. Increased production of proinflammatory cytokines by proband monocytes after stimulation with lipopolysaccharide.
**Özyurt *et al*. ** **(2019)**	60 drug-naïve adolescent OCD probands and 128 adolescent control subjects	Human cell models	Significant differences were detected in the neutrophil: lymphocyte ratio and white blood cell, neutrophil, and platelet counts among OCD probands with comorbid anxiety disorder, OCD probands with no comorbidities, and control subjects, which remained after controlling for age and sex.
**Hibar *et al*. ** **(2018)**	First OCD GWAS sample and neuroimaging GWASs of volume of eight subcortical brain regions (13,171 subjects)	Imaging genetics	OCD risk SNPs were associated with increased volumes of the nucleus accumbens and the putamen. Conditional false discovery rate analysis revealed specific SNPs influencing OCD risk and putamen, amygdala, and thalamus volumes.
**Zartaloudi *et al*.** ** (2019)**	707 unaffected relatives of OCD probands and 842 control subjects	Meta-analysis of endophenotype studies	21 studies were included in the meta-analysis. A significant impairment in global executive functioning was found among unaffected relatives of OCD probands, with specific deficits in planning, visuospatial working memory, and verbal fluency.
**Qin *et al*. (2016)**	804 OCD probands (514 responders and 290 non-responders)	Pharmacogenetics	A genome-wide significant SNP was detected near the gene *DISP1*, within a chromosomal region associated with neurodevelopment. Most significant SNPs were enriched for biological pathways related to glutamate and serotonin neurotransmission.
**Lisoway *et al*.** ** (2018)**	112 OCD probands	Pharmacogenetics	No significant associations between a SNP of *DISP1* and response to serotonin reuptake inhibitor treatment.

## Genome variants

Early studies on the genetics of OCD focused on candidate gene association studies^15^. Because of their insufficient power and biased *a priori* hypotheses, these studies produced rather inconsistent findings and are now considered obsolete^19^. The development of novel methods capable of assessing the entire genome, coupled with the recruitment of larger samples, allowed the undertaking of unbiased and powerful genetic investigations^19^. In this regard, cumulative evidence supports the idea that both common and rare genome variants contribute to the polygenic architecture of psychiatric disorders^20^. Accordingly, distinct methods have been recently used to detect genome variants associated with OCD, as described below.

### Common variants

**Figure 2.**
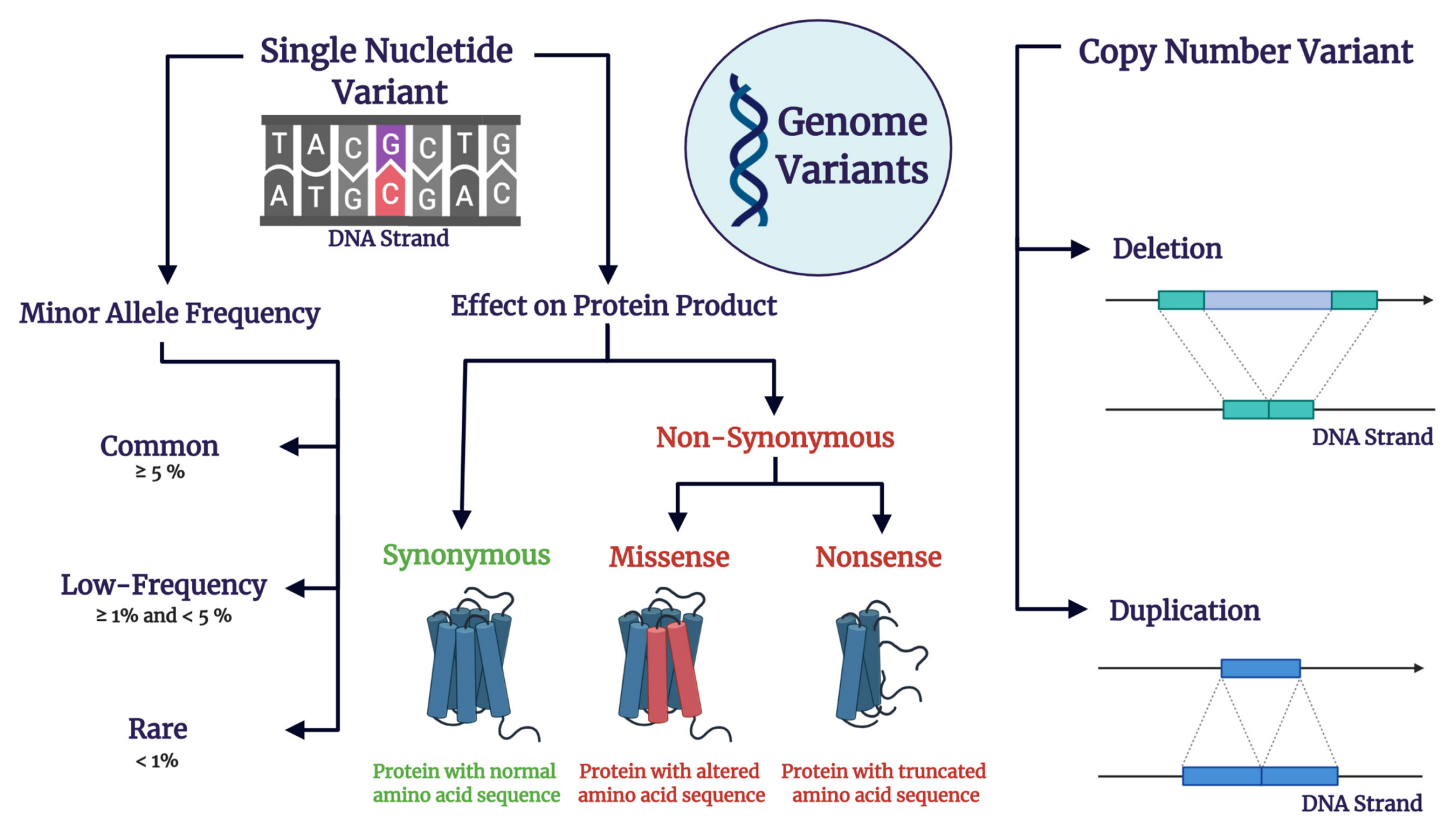
Variations in the genome. Single nucleotide variants (SNVs) constitute positions in the genome comprising a pair of bases for which different alleles (i.e. sequence variants) are found in a population. A SNV can be classified according to the frequency at which its second most common allele is found in a population, termed minor allele frequency (MAF). According to their MAF, SNVs can be classified into common (MAF≥5%), low-frequency (1%≥MAF<5%), and rare (MAF<1%)^18^. SNVs that occur in at least 1% of the population are called single nucleotide polymorphisms (SNPs)^18^. When occurring inside the exons (i.e. DNA stretches encoding protein products), SNVs can lead to the production of protein products with a normal amino acid sequence (i.e. synonymous SNVs), with an altered yet full amino acid sequence (i.e. missense SNVs), or with a truncated (i.e. incomplete) amino acid sequence (i.e. nonsense SNVs). The last two types of SNVs are termed nonsynonymous and usually have deleterious biological consequences. Copy number variants (CNVs) comprise genome deletions and duplications.

GWASs employ a case-control design to detect single nucleotide polymorphisms (SNPs) (Figure 2) associated with a disorder^21^, with significance thresholds set at *P* = 5 × 10^-8^^22^. A range of secondary analyses of GWAS data can be conducted to further probe the relevant genetic architecture^23^. Such analyses include determining the portion of heritability conferred by the SNPs investigated in a GWAS, defined as SNP-based heritability (*h*^2^_SNP_)^24^, estimating the degree of shared genetic architecture between disorders by determining their genetic correlation^25,26^, calculating PRSs based on risk variants for a disorder identified in a GWAS^27^, and undertaking enrichment analysis to assess clustering of detected variants within functionally related genomic regions and biological pathways^28^.

To date, GWASs of OCD have not revealed any genome-wide significant SNPs associated with this disorder in case-control analyses^15^. Recently, the Psychiatric Genomics Consortium OCD (PGC-OCD) workgroup combined data from the first two OCD GWASs^29,30^ to yield a sample of 2,688 OCD cases and 7,037 controls. The top-ranked detected SNP (i.e. strongest association) was in the *P* = 10^-7^ significance range^31^. Nonetheless, SNP-based heritability was estimated at 28% in this sample^31^, among the highest reported for neuropsychiatric disorders. These findings indicate the need for still larger samples for sufficient statistical power.

The top-ranked SNPs detected in the PGC-OCD GWAS were in linkage disequilibrium with genes previously associated with autism spectrum disorders (ASD)^31^: *GRID2*, involved in cerebellar synaptic processes^32^, and *KIT*, implicated in neurodevelopment^33^. Moreover, a sex-stratified GWAS of the PGC-OCD sample revealed that SNPs with sex-specific effects were significantly enriched for genome regions influencing gene expression (i.e. expression quantitative trait loci, eQTLs) in brain and immune tissues^34^. In line with the findings of an earlier GWAS of obsessive-compulsive symptoms^35^, a recent meta-analysis of the PGC-OCD GWAS and a GWAS of compulsive symptoms in 8,267 nonclinical subjects revealed the most significant enrichment for genes expressed in brain regions associated with the neurocircuitry of OCD, i.e. the cingulate cortex, nucleus accumbens, and amygdala^36^. Taken together, the aforementioned GWASs suggest a role for synaptic, neurodevelopmental, and immune processes in the etiology of OCD.

Genetic correlation analyses using data from the PGC-OCD GWAS have suggested a shared genetic architecture between OCD and related conditions. In this respect, two studies found a significant genetic correlation between OCD and anorexia nervosa (AN)^37,38^, consistent with the hypothesis that obsessive and compulsive traits in these conditions have shared etiology^39^. Furthermore, PRSs for other neuropsychiatric disorders were able to significantly predict case-control status in previous OCD GWAS samples, indicating a shared genetic liability^40^. In smaller studies, a PRS for schizophrenia (SCZ) significantly predicted case-control status in a Spanish sample comprising 370 OCD patients and 443 controls^41^, and a PRS for ASD significantly predicted case-control status in a sample of 2,535 OCD cases and controls^42^. PRS may, in the future, have applications in clinical practice, ranging from improving diagnostic precision to predicting treatment response^20^. However, it needs to be emphasized that such applications are not currently possible and that translation of PRSs from a population of one ancestry to a population of different ancestry is problematic^43^.

### Rare variants

WES is a technique employed to detect single nucleotide variants in the exome (i.e. all of the genome exons)^20^, among which *de novo* variants (Figure 2) have the most pathogenic effect for neuropsychiatric disorders^44^. *De novo* variants refer to genome variants that occur in the proband but not in the parents. WES studies are usually performed in trios composed of healthy parents and an affected child in order to detect *de novo* variants driving pathology^20,44^.

Two recent studies reported an increased genome-wide burden of gene-disrupting *de novo* mutations in patients with OCD. In the first WES study^45^, 20 nonsynonymous *de novo* variants were detected among 17 OCD probands at a rate similar to that reported for other neuropsychiatric disorders. Moreover, these variants were enriched for pathways related to the immune system and neurodevelopment.

The second WES study was conducted on a sample of 184 OCD and 777 control parent–child trios^46^. *De novo* damaging variants were significantly more frequent among probands, achieving a prevalence of 33.9%. Such findings were used to estimate the rate of these variants among overall OCD cases at 22.2%. Two high-confidence risk genes for OCD were further identified: *SCUBE1*, potentially involved in endothelial inflammatory responses^47^, and *CHD8*, implicated in chromatin metabolism^48^, neurogenesis^49^, and synaptic transmission^50^ and extensively associated with ASD^51–53^. Additionally, genes carrying *de novo* damaging variants in OCD probands significantly overlapped with genes associated with ASD, Tourette’s syndrome (TS), and other neurodevelopmental disorders and were enriched for immune system pathways. Similar findings were reported in another study which found a significant overlap between genes associated with OCD and genes associated with ASD; in addition, the overlapping genes were significantly enriched for biological pathways related to brain function^54^.

### Copy number variants

The most common type of structural variants in the human genome are CNVs (Figure 2), which comprise duplications and deletions^55^. The prevalence of CNVs can be assessed across the whole genome using array methods^56^.

In an earlier CNV study in OCD, a 4.4-fold increase in deletions encompassing loci associated with neurodevelopmental disorders (including 16p13.11) was found among OCD probands^57^. More recently, two CNV studies were conducted on samples comprising 307 and 121 individuals with pediatric OCD and 3,861 and 124 healthy controls, respectively^58,59^. The number of CNVs in genes involved in brain function was higher among OCD probands in both studies. In the first study, CNVs were found in three targets of the fragile X mental retardation protein (FMRP). Consistent with FMRP involvement in several processes related to synaptic plasticity^60^, CNVs encompassing FMRP targets have been associated with SCZ^61^, ASD^62^, and attention deficit hyperactivity disorder (ADHD)^63^. In the second study, CNVs were found in genes implicated in SCZ, ASD, and TS. Finally, a recent study of 2,691 individuals diagnosed with OCD, SCZ, ASD, or ADHD found clinically relevant CNVs in 5.6% of OCD cases and multiple brain-expressed genes impacted by CNVs overlapping across those disorders^64^.

Taken together, these WES and CNV findings again support a role for synaptic, neurodevelopmental, and immune processes in the etiology of OCD. Moreover, these findings indicate the existence of a shared genetic etiology between OCD and related disorders, suggesting that their current diagnostic categories may not reflect distinct clinical entities. In line with this, a recent study used a machine learning approach to cluster patients with OCD, ASD, and ADHD into homogenous groups based on neuroimaging measures of cortical thickness and behavioral measures and found that those diagnostic categories were not reflected in the groups formed^65^.

## Variation in gene expression

Complex regulatory mechanisms govern the way in which genotype and environment together lead to distinctive patterns of gene expression in different tissues at different developmental stages^20,66,67^. Methods are now available to explore tissue-specific gene expression and environmental regulatory mechanisms in OCD.

### Gene expression in specific tissue

**Figure 3.**
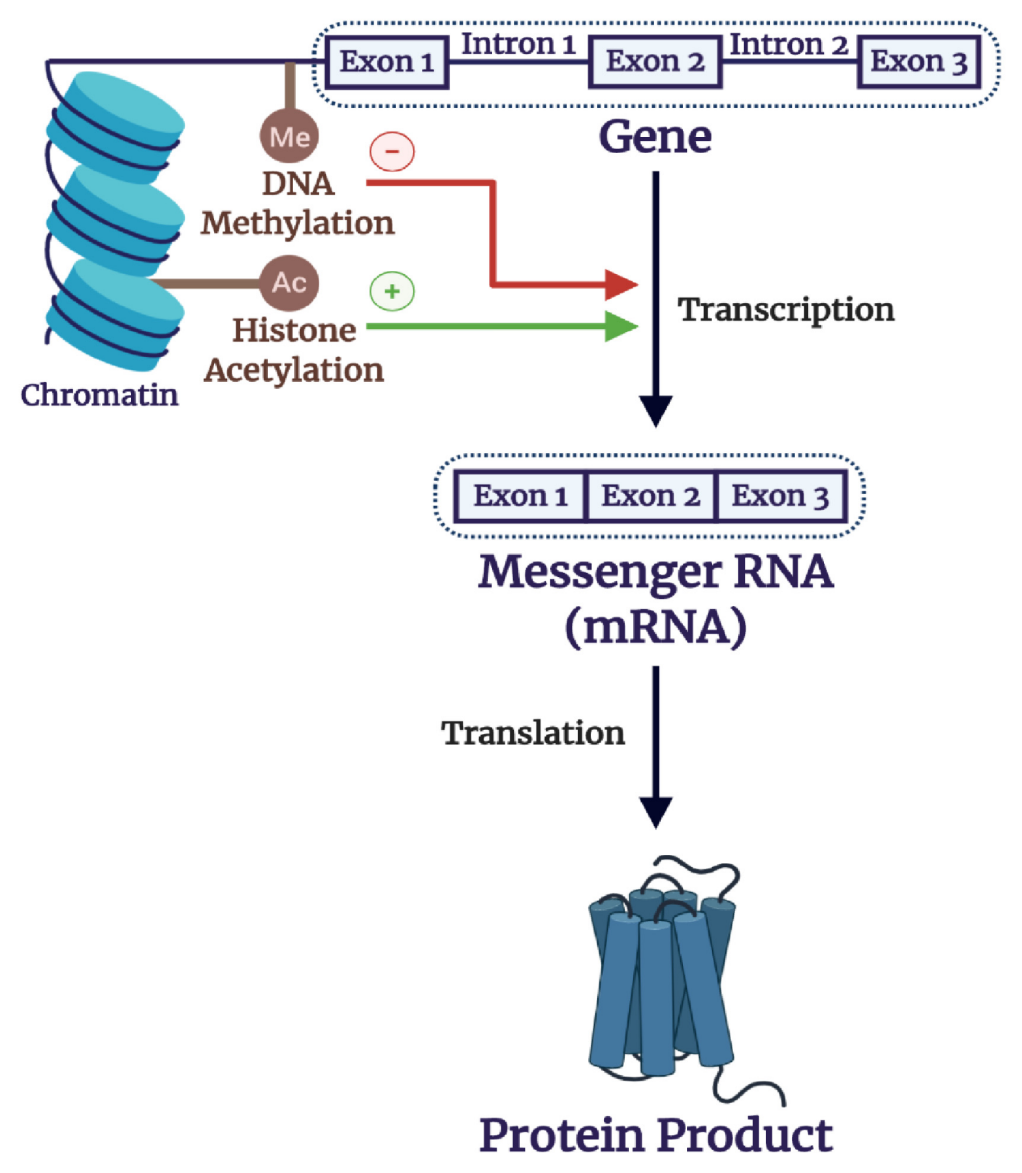
Gene expression. The gene, composed of exons (protein-coding regions) and introns (non-protein-coding regions), is transcribed into a precursor messenger ribonucleic acid (pre-mRNA), which undergoes alternative splicing to generate a messenger RNA (mRNA), called a primary transcript. Finally, mRNA undergoes translation to generate a protein product. This chain of events enables the flow of genetic information.

As a proxy of gene expression, the measurement of transcript levels (i.e. mRNA) (Figure 3) in post-mortem brain specimens of patients with psychiatric disorders is useful for genetic investigations^68^. Furthermore, the discovery of differential transcript levels in the peripheral blood of patients with psychiatric disorders may provide additional insight into mechanisms underlying gene expression^69^.

Recently, reduced expression of genes related to excitatory glutamatergic synapses has been identified in the lateral and frontal regions of the orbitofrontal cortex (OFC) of individuals with OCD^70^. Speculatively, such findings may be relevant to understanding alterations in OFC volume^71^ and activity in neuroimaging studies of OCD^72^.

Gene expression profiling in the peripheral blood has been employed to compare individuals with OCD, major depressive disorder (MDD), and SCZ and healthy controls^73^. A six-gene panel was able to diagnose OCD cases with 88% sensitivity and 85% specificity^73^. Among the genes included in the panel, *FKBP1A* encodes an enzyme involved in immune regulation^74^ and *COPS7A* encodes a subunit of a protein complex involved in protein degradation^75^.

### Gene expression and the environment

The reciprocal interactions between gene expression and the environment have been investigated through diverse methods, ranging from basic science to epidemiological approaches^76,77^. Importantly, epigenetic mechanisms (Figure 3) provide a pathway whereby the environment can modulate gene expression through alterations in chromatin and DNA structure^78^.

In one recent study, no difference was found in blood DNA methylation levels of candidate genes previously implicated in OCD among 21 pediatric cases and 12 controls^79^. Conversely, in another study using a genome-wide approach, multiple genes with different methylation levels were detected among 65 pediatric OCD cases and 96 controls^80^. These findings emphasize the importance of an unbiased approach for studying the genomics of OCD.

A study of gene–environment interactions in a cohort of 103 patients with OCD revealed that neither PRS for OCD nor the presence of a stressful life event at the onset of the disorder predicted treatment response^81^. Nonetheless, PRS was able to predict illness severity. Moreover, environmental factors, such as perinatal complications^82,83^ and maternal effects (i.e. the influence of maternal genetic and environmental factors on the phenotype of the offspring)^84^, have been shown to increase the risk for OCD. Further work is needed to determine whether PRSs, taken together with a range of environmental factors, may be useful in predicting OCD severity and treatment response. As a complement to the gene–environment interaction approach, in-depth environmental research in psychiatry, known as the exposome^85^, should add to the understanding of the interplay between genetics and the exposure to multiple internal and external stimuli in the prevention and treatment of psychiatric disorders.

## Modeling genetic architecture

Laboratory-based experimental models may be used to study genes which appear to be important in clinical studies of the genomics of OCD^86^. We focus here on work done in animals and in human cells.

### Experimental animal systems

Excessive grooming, hoarding behaviors (e.g. marble-burying), and stereotyped behaviors have been studied in rodent models of OCD^87^. Such work is not intended to replicate the disease in an animal system but may rather provide insight into the biological mechanisms relevant to OCD, which may foster the development of novel treatment approaches^88^.

Overexpression of the excitatory amino acid transporter 3 (EAAT3), involved in glutamate neurotransmission, in the frontal cortex, hippocampus, and striatum of rodents resulted in increasing grooming and disturbed cortical–striatal excitatory synapse plasticity^89^. Furthermore, a protective effect against the induction of stereotyped behaviors by dopaminergic challenge was obtained by constitutive EAAT3 reduced expression in mice^90^.

Immunity- and neuroinflammation-related processes have been consistently associated with OCD^91^. In this respect, mice with microglia-restricted progranulin inactivation exhibited increased self-grooming and marble-burying, which was normalized after suppression of nuclear factor κB signaling in the microglia^92^. Also suggesting a potential role for the microglia in the etiology of OCD, targeted deletion of Hoxb8-lineage microglia yielded severe grooming, especially in female mice^93^. Furthermore, restoring tropomyosin receptor kinase B/ERK-MAPK signaling in mice** normalized severe grooming behaviors induced by ablation of *SPRED2*^94^, which pertain to a family of proteins that has been implicated in neurodevelopment^95^.

Recent investigations of gene pathway analysis^96^ and PRSs^97^ using GWAS data support the association of disturbances in insulin signaling with the pathophysiology of OCD. These findings were further validated in a rodent model of type 2 diabetes mellitus, which revealed increased compulsive-like behaviors and brain abnormalities previously associated with OCD^98^.

Findings from experimental animal systems may inform clinical studies^99^. A large set of candidate genes potentially associated with OCD in previous human, rodent, and canine studies was investigated using a target-sequencing approach for the detection of regulatory and coding variants (i.e. functional variants) in 592 OCD cases and 560 controls^100^. Among the four genes significantly enriched for functional variants in OCD cases (i.e. *NRXN1*, *HTR2A*, *CTTNBP2*, and *REEP3*), all were involved in brain biological pathways implicated in OCD^100^. Additionally, *NRXN1* achieved genome-wide significance when OCD cases were compared to 33,370 controls^100^.

### Human cell models

Investigations using human-derived cells as models may be useful in studying OCD at the molecular and cellular levels^101^.** Higher ratios of immune cells in the peripheral blood^102,103^ and abnormal production of inflammatory cytokines upon stimulation by those cells^102^ have been reported in OCD. Future work on pluripotent stem cells derived from somatic cells of patients with OCD could be useful in extending this preliminary work.

## Genetic variations underlying relevant phenotypes

There is growing interest in determining the genetic basis of OCD-associated neuroimaging abnormalities, cognitive dysfunction, and treatment response, as outlined below.

### Neuroimaging

The Enhancing Neuroimaging Genetics through Meta-Analysis (ENIGMA) consortium is the largest collaboration working to integrate genetic and neuroimaging findings; it combines findings from sites across the globe^104^.** The combination of data from the first OCD GWAS and the ENIGMA GWAS of subcortical brain structure^105^ allowed for the detection of a significant overlap between the SNPs associated with increases in the risk for OCD and in the volumes of the putamen, a component of the cortical–striatal–thalamic–cortical circuitry implicated in OCD^106^, and the nucleus accumbens, a treatment target for deep brain stimulation in treatment-refractory OCD^107^. Moreover, the SNP associated with both the increased risk for OCD and the increased putamen volume is located near the *RSPO4*, a gene implicated in pathways related to neurodevelopment^108^.

### Cognitive dysfunction

Cognitive deficits have been frequently explored as OCD endophenotypes^109^, defined as heritable quantitative traits found at higher rates in unaffected relatives of patients and associated with increased genetic susceptibility to the disorder^110,111^. The findings of investigations on cognitive deficits as endophenotypes of OCD have been recently meta-analyzed^112^; this study found significant impairment in global executive functioning among unaffected relatives, with specific deficits in planning, visuospatial working memory, and verbal fluency. Since these endophenotypes may be more proximally related to genetic mechanisms than OCD itself^111^, further investigation of these deficits may be useful.

### Treatment response

Pharmacogenetic studies address the association of genetic variation with drug response^113^. Although earlier pharmacogenetic studies in psychiatric disorders have focused mainly on candidate genes and on genes related to the cytochrome P450 system^114^, genome-wide approaches have been recently employed in OCD research.

The association of the response to serotonin reuptake inhibitors (SRIs) treatment with common variants was assessed in a GWAS of 804 OCD cases, including 514 responders and 290 non-responders^115^. A genome-wide significant SNP was detected near the gene *DISP1*, within a chromosomal region associated with neurodevelopment. Further enrichment analysis revealed that the most significant SNPs were enriched for biological pathways related to glutamate and serotonin neurotransmission. However, a follow-up study with 112 OCD cases found no association between the SNP located near *DISP1* and the response to SRIs treatment^116^. Further investigation in larger samples is warranted to determine the clinical utility of pharmacogenetic data in OCD.

## OCD genetic architecture: an exploratory model

**Figure 4.**
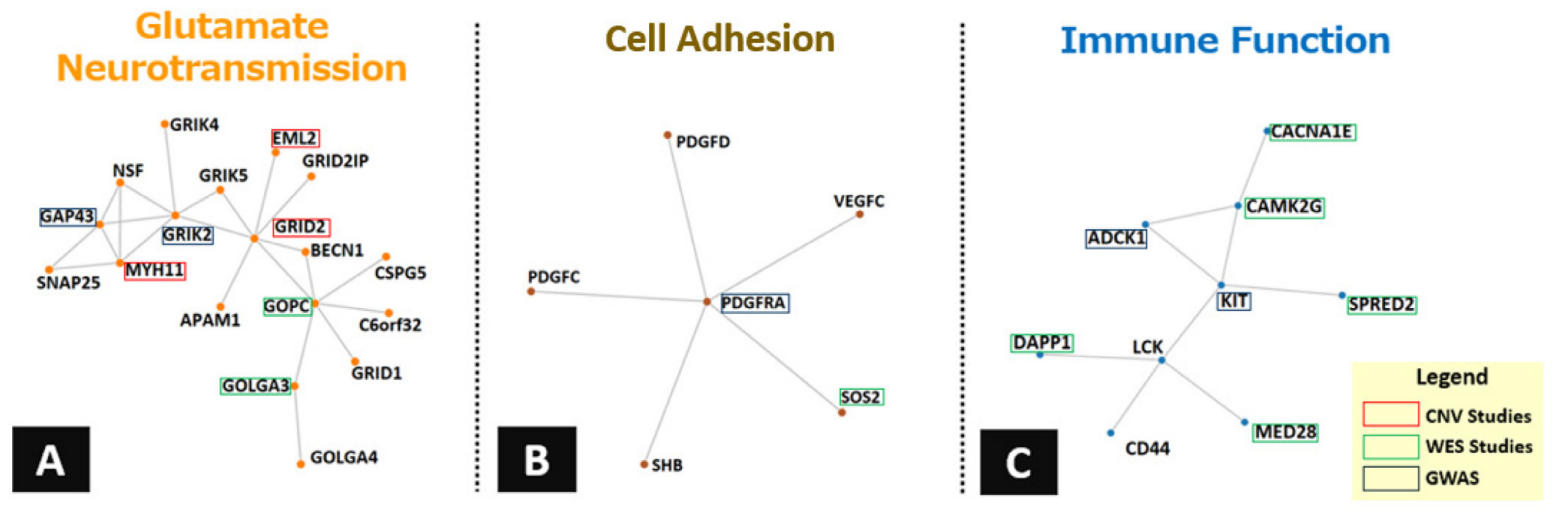
Gene networks exploratory analysis. The GeneNets algorithm was used to ascertain whether a set of obsessive-compulsive disorder (OCD) risk genes were more significantly connected to each other in a functional network as would be expected by chance alone. Accordingly, a total of 204 OCD-associated genes were selected from studies cited throughout the present review (more specifically, seven genome-wide association studies [GWASs]^29–31,35,36,41,42^, two whole-exome sequencing [WES] studies^45,46^, two copy number variant [CNV] studies^58,59^, and one functional variants study^100^) for the GeneNets exploratory analysis. A total of 125 of these genes were included in a significant network, in which three communities were enriched for biological pathways. More specifically, the most significant pathways detected for those communities were the ionotropic activity of kainate receptors (*P* <10^-6^), associated with glutamate neurotransmission^118^ (Figure 4A); the cell adhesion-related processes (*P* <10^-9^), involved in synaptic processes^119^ and neurodevelopment^120,121^ (Figure 4B); the regulation of kit signaling (*P* <10^-4^), associated with immune function^122^ (Figure 4C); and the activation of GABAA receptors (*P* <10^-12^), implicated in neuropsychiatric disorders^123^. For the pathways displayed in the figure, the type of study from which their respective genes were selected is highlighted in the figure.

The data described in the previous sections suggest that genes involved in synaptic transmission, neurodevelopment, and the immune and inflammatory systems are involved in the pathophysiology of OCD (Figure 1). Based on these data, an exploratory analysis was undertaken with GeneNets in order to investigate whether genes associated with risk for OCD, identified in studies assessing common and rare variants, would form communities (i.e. clusters of functionally connected genes involved in a particular biological process)^117^. Three communities were significantly enriched for biological processes: related to glutamate neurotransmission, to cell adhesion, and to the immune system (Figure 4). These findings are consistent with a model in which multiple genes and biological pathways play a role in the pathogenesis of OCD and in which synaptic, neurodevelopmental, and immune pathways may be particularly important.

## OCD genetics: current insights and future prospects

This review shows how several recent developments in genomics have contributed to understanding the genetic architecture of OCD. The use of cutting-edge methods has moved the field from a focus on candidate genes in underpowered samples to unbiased approaches in larger cohorts. At the same time, a great deal more work is needed to fully understand the role of common and rare gene variants in OCD. Work on gene methylation and expression has provided proof-of-principle demonstrations of the value of such studies and deserves expansion. Such work, taken together with careful phenotyping and comparison of individuals with different disorders, may lead to a better understanding of transdiagnostic genetic mechanisms.

The question of how advances in the genomics of OCD will become clinically useful remains to be answered. First-line pharmacological treatments for OCD target mostly serotonergic neurotransmission; biological pathways implicated in recent genomic studies, such as those related to glutamatergic, neurodevelopmental, and the immune and inflammatory systems, may be useful targets in the future. More robust pharmacogenetic evidence may ultimately improve the prediction of response to pharmacological treatments, advancing precision medicine for OCD. Shared genetic liability among OCD, ASD, and SCZ may suggest new approaches to transdiagnostic assessment of patients. That said, to date, genetic studies have not demonstrated clinical utility, suggesting that OCD is not a simple homogeneous entity but rather that the underlying biological pathways are complex, variable, and heterogeneous. We can expect that translation from bench to bedside, through continuous effort and collaborative work, will ultimately transform our understanding of what causes OCD and thus how best to treat it.
